# Whole-genome sequencing of multidrug-resistant *Mycobacterium tuberculosis* isolates from Myanmar

**DOI:** 10.1016/j.jgar.2016.04.008

**Published:** 2016-09

**Authors:** Htin Lin Aung, Thanda Tun, Danesh Moradigaravand, Claudio U. Köser, Wint Wint Nyunt, Si Thu Aung, Thandar Lwin, Kyi Kyi Thinn, John A. Crump, Julian Parkhill, Sharon J. Peacock, Gregory M. Cook, Philip C. Hill

**Affiliations:** a Department of Microbiology and Immunology, Otago School of Medical Sciences, University of Otago, Dunedin, New Zealand; b Maurice Wilkins Centre for Molecular Biodiscovery, University of Auckland, Auckland, New Zealand; c National Health Laboratory, Ministry of Health, Yangon, Myanmar; d Department of Medicine, University of Cambridge, Cambridge, UK; e National TB Programme (NTP), Ministry of Health, Naypyidaw, Myanmar; f Department of Microbiology, University of Medicine 1, Yangon, Myanmar; g Centre for International Health, Dunedin School of Medicine, University of Otago, Dunedin, New Zealand; h Wellcome Trust Sanger Institute, Wellcome Trust Genome Campus, Hinxton, UK; i London School of Hygiene and Tropical Medicine, London, UK

**Keywords:** Whole-genome sequencing, Drug-resistant TB, Myanmar

## Abstract

Drug-resistant tuberculosis (TB) is a major health threat in Myanmar. An initial study was conducted to explore the potential utility of whole-genome sequencing (WGS) for the diagnosis and management of drug-resistant TB in Myanmar. Fourteen multidrug-resistant *Mycobacterium tuberculosis* isolates were sequenced. Known resistance genes for a total of nine antibiotics commonly used in the treatment of drug-susceptible and multidrug-resistant TB (MDR-TB) in Myanmar were interrogated through WGS. All 14 isolates were MDR-TB, consistent with the results of phenotypic drug susceptibility testing (DST), and the Beijing lineage predominated. Based on the results of WGS, 9 of the 14 isolates were potentially resistant to at least one of the drugs used in the standard MDR-TB regimen but for which phenotypic DST is not conducted in Myanmar. This study highlights a need for the introduction of second-line DST as part of routine TB diagnosis in Myanmar as well as new classes of TB drugs to construct effective regimens.

## Highlights

• Drug-resistant tuberculosis (TB) is a major health threat in Myanmar.
• The first whole-genome sequencing study of drug-resistant TB from Myanmar.
• Introduction of second-line drug susceptibility testing as part of routine diagnosis in Myanmar is needed.

## 1 Introduction

Myanmar is one of the 22 high-burden tuberculosis (TB) countries, with a high prevalence of multidrug-resistant TB (MDR-TB) [1]. Rapid detection is essential to treat patients with drug-resistant TB. Yet conventional drug susceptibility testing (DST) takes several weeks owing to the culturing requirement and subsequent laborious phenotypic testing. Consequently, molecular DST using the Hain GenoType MTBDR*plus* v.2.0 (Hain Lifescience GmbH, Nehren, Germany) and, more recently, the Cepheid GeneXpert MTB/RIF (Cepheid, Sunnyvale, CA) has been established in Myanmar. However, these assays only interrogate the most frequent resistance mutations for a limited number of antibiotics. Whole-genome sequencing (WGS) has the potential to overcome this limitation and can be used to identify patients with drug-resistant TB [2], [3], [4]. Whilst WGS is being considered for routine diagnosis and management of drug-resistant TB in well-resourced, low-TB burden settings, currently there are no plans for routine implementation in resource-limited, high-TB burden countries. Since it is important that new tools with the potential to improve TB control are adopted as early as possible especially in countries where these tools are needed the most, a preliminary evaluation of the utility of WGS in the diagnosis and management of drug-resistant TB in Myanmar was conducted.

## 2 Materials and methods

According to Myanmar national guidelines for the management of MDR-TB [5], suspected MDR-TB patients (Table 1) who are sputum smear-positive are referred to the National TB Reference Laboratories in Yangon and Mandalay for genotypic testing with the Hain GenoType MTBDR*plus* v.2.0 as well as phenotypic DST. Sputum specimens are decontaminated and are then inoculated onto Löwenstein–Jensen medium for culturing and phenotypic DST of isoniazid, rifampicin, ethambutol and streptomycin [6]. The MTBDR*plus* is performed according to the manufacturer's instructions and DNA is extracted as described previously [7]. The National Reference Laboratories in Myanmar do not currently perform DST of second-line drugs or pyrazinamide as part of routine diagnosis of drug-resistant TB and do not store culture isolates.

DNA of 14 isolates from MDR-TB patients were selected for this study and were further purified using an UltraClean^®^ Microbial DNA Isolation Kit (Mo Bio Laboratories, Carlsbad, CA). DNA was sequenced using paired-end 250-bp reads on an Illumina MiSeq using the Nextera™ XT DNA Kit (Illumina Inc., Hayward, CA). The resulting sequencing data were submitted to the European Nucleotide Archive (PRJEB10037). Using version 1 of PhyResSE with version 27 of the variant list and, where applicable, literature review, resistance genes for the following antibiotics that are commonly used in the treatment of drug-susceptible and MDR-TB in Myanmar were interrogated: rifampicin; isoniazid; ethambutol; streptomycin; ethionamide; pyrazinamide; amikacin; and levofloxacin [8]. In addition, genes involved in para-aminosalicylic acid (PAS) resistance (*folC*, *ribD* and *thyA*) were analysed [2]. PhyResSE was also used for strain classification.

## 3 Results

### 3.1 Strain diversity

The Beijing lineage dominated, with 11 of the 14 strains belonging to that particular lineage. The remaining three strains belonged to the East-African Indian and Euro-American lineages (Table 2).

### 3.2 Genotype–phenotype concordance for drugs that were tested phenotypically in Myanmar

The WGS results for *rpoB*, *inhA* and *katG* were in full agreement with the results of the MTBDR*plus* (Table 2). Moreover, two *katG* mutations were detected in isoniazid-resistant strains that cannot be detected with the MTBDR*plus*: G299C in M00011, which is known to be associated with isoniazid resistance; and a frameshift in M00020, which should result in high-level isoniazid resistance [9].

Of the 14 strains, 7 were phenotypically resistant to ethambutol and harboured known resistance mutations in *embB* or mutations that were previously associated with ethambutol resistance. One phenotypically susceptible strain (M00004) had a known resistance mutation [4], [10].

With respect to streptomycin, all but one strain was phenotypically resistant, but genotypic resistance was identified in only 10 of the 13 resistant strains, due to mutations in *rpsL* that are known to be associated with streptomycin resistance or to potential resistance mutations in *gidB* [4]. It was not possible to test whether these discrepancies were due to laboratory error, given that the strains were not stored.

### 3.3 Genotypic drug susceptibility testing for drugs used in multidrug-resistant tuberculosis treatment

No phenotypic DST results were available for ethionamide, pyrazinamide, amikacin, levofloxacin, PAS and cycloserine. In addition to the two *inhA* mutants, two strains were identified that were likely ethionamide-resistant as a result of a previously described *ethA* mutation [11]. Of the 14 strains, 6 had mutations that are known to be associated with pyrazinamide resistance [12]. Strain M00017 was predicted to be resistant to amikacin as a result of an *rrs* G1484T mutation [13]. Two strains had high-confidence *gyrA* resistance mutations to levofloxacin [14]. Strain M00005 was most likely resistant to PAS as a result of a premature stop codon in *thyA* [15]. Three more strains were potentially resistant to PAS owing to novel mutations in *folC* or *thyA*. No genotypic prediction for cycloserine was performed as the genotypic basis of resistance is poorly understood.

## 4 Discussion

This is the first study to report WGS data for drug-resistant TB from Myanmar and provides possibilities for incorporating WGS into clinical management of drug-resistant TB in Myanmar. For example, WGS can provide a diagnosis of resistance to multiple drugs more quickly than standard phenotypic DST so that it can be used to guide treatment of highly drug-resistant cases such as extensively drug-resistant TB (XDR-TB) cases. It can also serve as a tool for quality control to monitor laboratory performance. Furthermore, it could be used to understand transmission in a population. A larger study is planned in Myanmar to explore these possibilities given that sequencing costs are reducing rapidly (now less than US$200 per *Mycobacterium tuberculosis* isolate) and the availability of fully automated analysis is underway.

Predominance of the Beijing lineage in this study confirmed prior findings of MDR-TB in Myanmar [16]. Isoniazid and rifampicin resistance was primarily due to mutations in codon 315 of *katG* and in codon 531 of *rpoB*, respectively, as previously observed [17], [18]. The detection of rare mutations in *katG* that cannot be detected with the MTBDR*plus* highlights the added value of WGS to resolve discrepancies between phenotypic and genotypic results. The discordance between the genotype and phenotypic ethambutol results in this study was in line with previous findings that ethambutol DST is less reproducible than for other first-line drugs [4].

The drugs used in the MDR-TB regimen in Myanmar consist of 6 months of amikacin, pyrazinamide, levofloxacin, ethionamide and cycloserine, followed by 18 months of pyrazinamide, levofloxacin, ethionamide and cycloserine (and PAS if ethionamide resistance is detected) (Table 1) [5]. None of the strains in this study were predicted to be XDR-TB. However, 9 of the 14 isolates were likely resistant to at least one of the drugs in the aforementioned standard MDR-TB regimen. Moreover, four of the strains (M00004, M00013, M00017 and M00019) were likely resistant to two drugs, which would reduce the number of effective drugs to three in the intense phase and to two during the extended phase (three in the case of M00017). For the strains with *gyrA* mutations, resistance to levofloxacin might have been overcome by replacing levofloxacin with moxifloxacin, to which these mutations generally confer low-level resistance [14]. By contrast, adding PAS to the regimen of the two potentially ethionamide-resistant strains with *ethA* M1R mutations (according to standard treatment guidelines) might not have been effective if the *folC* mutations in both strains also caused resistance. Replacing amikacin with kanamycin or capreomycin in the patient with the *rrs* mutation would not have been an option as this mutation confers cross-resistance to all of these aminoglycosides [13]. Consequently, novel classes of TB drugs are required in Myanmar to construct appropriate regimens.

This study highlights the need to introduce second-line DST in routine diagnosis in Myanmar to substantially increase the proportion of MDR-TB patients for whom DST is conducted (in 2013, only 4.4% of confirmed MDR-TB cases were tested for a fluoroquinolone and second-line injectable drug) [1]. This could also provide clarity regarding the prevalence of XDR-TB, which is currently unknown despite the first reported case in 2007 [19]. This could be achieved using phenotypic methods or by introducing one of the current commercial genotypic DST assays such as AID Resistance Module FQ/EMB and STR/AMK/CAP (AID Diagnostika GmbH, Straßberg, Germany), MTBDR*sl* v.1.0 or 2.0 (Hain Lifescience GmbH) and the Nipro FQ and PZA line probe assay (LiPA) (Nipro Corp., Osaka, Japan) [20]. It should be noted, however, that their ability to detect low-level heteroresistance is poorly understood. Moreover, LiPAs are relatively labour intensive and slow. More decentralised testing for XDR-TB would be preferable (e.g. with the XDR cartridge that is currently being developed for the GeneXpert and GeneXpert Omni). At the same time, this study revealed that even if a prompt and accurate DST service is introduced, new classes of TB drugs are urgently required in Myanmar to construct regimens that are sufficiently active to adequately treat drug-resistant TB cases.

## Funding

The cost for whole-genome sequencing (WGS) was supported by the Maurice Wilkins Centre for Molecular Biodiscovery (Auckland, New Zealand). HLA was supported by a Professor Sandy Smith Memorial Scholarship to perform data analysis at the University of Cambridge and the Wellcome Trust Sanger Institute (Cambridge, UK). This publication presents independent research supported by the Health Innovation Challenge Fund [HICF-T5-342 and WT098600], a parallel funding partnership between the UK Department of Health and the Wellcome Trust. The views expressed in this publication are those of the authors and are not necessarily those of the Department of Health, Public Health England, or the Wellcome Trust. CUK is a Junior Research Fellow at Wolfson College, University of Cambridge.

## Competing interests

CUK, JP and SJP have collaborated with Illumina Inc. on a number of scientific projects; CUK is a consultant for the Foundation for Innovative New Diagnostics and a technical advisor for the Tuberculosis Guideline Development Group of the World Health Organisation. The Bill & Melinda Gates Foundation and Janssen Pharmaceutica covered CUK's travel and accommodation to present at meetings. The European Society of Mycobacteriology awarded CUK the Gertrud Meissner Award, which is sponsored by Hain Lifescience. JP has received funding for travel and accommodation from Pacific Biosciences Inc. and Illumina Inc. SJP has received funding for travel and accommodation from Illumina Inc. All other authors declare no competing interests.

## Ethical approval

Ethical approval for this study was given by the Research and Ethical Committee of the University of Medicine 1 (Yangon, Myanmar).

## Figures and Tables

**Table 1:** Treatment category by type of tuberculosis (TB) patient and recommended treatment regimens [5].

Treatment category	
Cat I	Treatment for new patients with first-line anti-TB drugs (2 months of INH, RIF, EMB, PZA/4 months of INH, RIF)
Cat II	Re-treatment regimen with first-line anti-TB drugs (2 months of STR, INH, RIF, EMB, PZA/1 month of INH, RIF, EMB, PZA/3 months of INH, RIF, EMB)
New	A patient who has never had treatment for TB or who has taken anti-TB drugs for <1 month
Close contact	Close contacts of MDR-TB patients who develop active TB
Failure	A patient previously treated for TB who is started on a re-treatment regimen after previous treatment has failed
Relapse	A patient who was previously declared cured or treatment completed and is diagnosed with bacteriologically-positive (sputum smear or culture) TB
Standard MDR-TB regimen	6 months of AMK, PZA, LFX, ETH, DCS/18 months of PZA, LFX, ETH, DCS a

INH, isoniazid; RIF, rifampicin; EMB, ethambutol; PZA, pyrazinamide; STR, streptomycin; MDR, multidrug-resistant; AMK, amikacin; LFX, levofloxacin; ETH, ethionamide; DCS, cycloserine.

a Para-aminosalicylic acid will be added to the regimen if a resistance mutation in the *inhA* promoter is detected with MTBDR*plus* or if the patient cannot tolerate ETH or DCS.

**Table 2:** Summary of patient details and comparison of phenotypic drug susceptibility testing (DST) results, where available, with whole-genome sequencing (WGS) results.

ID	Age (years)	Sex	Type of patient	Genotype	RIF		INH		EMB		STR		ETH	PZA	AMK	LFX	PAS
					DST	*rpoB*	DST	Genotype	DST	*embB*	DST			*pncA*	*rrs*	*gyrA*	
M00001	55	F	Cat II, failure	Beijing	R	S531L	R	*katG* S315T	R	L402V	R	*rpsL* K43R a		W119G b			
M00003	28	M	Close contact (new)	Beijing	R	S531L	R	*katG* S315T	R	G406D	R	*rpsL* K43R a					
M00004	32	M	Cat II, failure	Beijing	R	S531L	R	*katG* S315T	S	M306I c, d	R	*rpsL* K43R a		FS b		A90V c, d, e, f	
M00005	36	F	Close contact (new)	Beijing	R	H526Y	R	*katG* S315T	R	G406A	R	*rpsL* K43R a		Q10P b			*thyA* W80*
M00008	75	M	Relapse after Cat II	Euro-American	R	H516V	R	*katG* S315T	S		R						
M00010	24	F	Cat II, failure	Beijing	R	S531L	R	*katG* S315T	S		R	*rpsL* K43R a					
M00011	63	M	Cat I, failure	Beijing	R	S531L	R	*katG* G299 g	R	M306V c, d	R					A90V c, d, e, f and D94A c, d, e, f	
M00012	44	M	Cat II, failure	East-African Indian	R	H526Y	R	*katG* S315T	S		R						*thyA* F176L h
M00013	19	M	Cat II, failure	Beijing	R	S531L	R	*katG* S315T	R	M306V c, d	R	*rpsL* K43R a	*ethA* M1R	Y103*^,^^b^			*folC* M54I h
M00016	48	M	Cat II, failure	East-African Indian	R	S531L	R	*inhA* C-15T	S		R	*gidB* E40G h	*inhA* C-15T				
M00017	68	M	Relapse after cat II	Beijing	R	S531L	R	*katG* S315T	R	E504D	R	*rpsL* K43R a		Q10P b	G1484T a, c, e		
M00018	63	F	Cat II, failure	Beijing	R	S531L	R	*katG* S315T	S		R	*gidB* P75T h					
M00019	27	M	Cat II, failure	Beijing	R	S531L	R	*katG* S315T	R	M306V c, d	R	*rpsL* K43R a	*ethA* M1R	T142A b			*folC* M54I h
M00020	42	M	Relapse after cat II	Beijing	R	S531L	R	*katG* FS g, *inhA* C-15T	S		S		*inhA* C-15T, *ethA* FS				

RIF, rifampicin; INH, isoniazid; EMB, ethambutol; STR, streptomycin; ETH, ethionamide; PZA, pyrazinamide; AMK, amikacin; LFX, levofloxacin; PAS, para-aminosalicylic acid; R, resistant; S, susceptible; FS, frameshift.

The Hain GenoType MTBDR*plus* v.2.0 (Hain Lifescience GmbH, Nehren, Germany) results for *rpoB*, *katG* and *inhA* were in agreement with the WGS data. Where applicable, alternative genotypic DST assays that could have been used to detect additional resistance mutations are listed, although it should be noted that low-level heteroresistant mutations might be below the detection limits of some of these assays, which are not well understood [20].

a Covered by AID TB Resistance Module STR/AMK/CAP (AID Diagnostika GmbH, Straßberg, Germany).

b Covered by Nipro PZA line probe assay (Nipro Corp., Osaka, Japan).

c Covered by Hain GenoType MTBDR*sl* v.1.0 (Hain Lifescience GmbH, Nehren, Germany).

d Covered by AID TB Resistance Module FQ/EMB (AID Diagnostika GmbH).

e Covered by Hain GenoType MTBDR*sl* v.2.0 (Hain Lifescience GmbH).

f Covered by Nipro FQ line probe assay (Nipro Corp.).

g Mutation not covered by MTBDR*plus*.

h Novel mutation with unknown effect.

* Stop codon.
